# Multi-Omics Biomarkers in Head and Neck Squamous Cell Carcinoma: Biological Insights and Clinical Translation: A Narrative Review

**DOI:** 10.3390/biology15151321

**Published:** 2026-08-06

**Authors:** Lucas de Araújo Albuquerque, Lilianny Querino Rocha de Oliveira, Déborah Gondim Lambert Moreira, Glória Maria de França, Roseana de Almeida Freitas, José Roberto Viana Silva, Everton Freitas de Morais

**Affiliations:** 1 Laboratory of Biotechnology and Physiology of Reproduction, Federal University of Ceara, Sobral 62041-040, CE, Brazil; lucas9521@alu.ufc.br (L.d.A.A.); jrvsilva@ufc.br (J.R.V.S.); 2 Laboratory of Nutrient and Tissue Repair (LABNUTRE), School of Applied Sciences, University of Campinas, Limeira 13484-350, SP, Brazil; l265902@dac.unicamp.br; 3 Department of Oral Pathology, Federal University of Rio Grande do Norte, Natal 59056-000, RN, Brazil; deborah.gondim.093@ufrn.edu.br (D.G.L.M.); roseana.freitas@ufrn.br (R.d.A.F.); 4 Professional Master’s Program in Health Research, Cesmac University Center, Maceió 57051-160, AL, Brazil; gloria.franca@cesmac.edu.br; 5 Department of Oral Diagnosis, School of Dentistry, University of Campinas, Piracicaba 13414-018, SP, Brazil

**Keywords:** squamous cell carcinoma, multi-omics, biomarkers, tumor microenvironment, molecular heterogeneity

## Abstract

Head and neck squamous cell carcinoma (HNSCC) continues to be an important health problem worldwide, with high morbidity and mortality and considerable biological variability among tumors. Although clinicopathological features remain essential in clinical practice, they do not fully explain differences in tumor behavior or treatment response. The growing availability of molecular data has therefore increased interest in multi-omics strategies for HNSCC. Genomic, transcriptomic, epigenomic, proteomic, and metabolomic analyses provide different, but complementary, views of tumor biology. Genomic alterations, including recurrent changes in *TP53* and *PIK3CA*, help define the molecular landscape of HNSCC, while transcriptomic biomarkers such as PD-L1 expression and epithelial–mesenchymal transition (EMT)-related signatures provide insights into tumor–microenvironment interactions, immune evasion, and treatment resistance. Epigenetic alterations further regulate gene expression, whereas proteomic and metabolomic biomarkers provide information more closely related to cellular function, metabolic reprogramming, and disease progression. More recently, single-cell and spatial approaches have allowed tumors to be studied according to their cellular composition and tissue organization. Artificial intelligence and machine learning are also being explored to combine these large datasets and identify biomarkers related to prognosis or therapeutic response. This review critically discusses the principal multi-omics biomarkers investigated in HNSCC, including biomarkers associated with genomic instability, immune regulation, EMT, and metabolic reprogramming, highlighting their potential clinical applications and the current barriers limiting their incorporation into routine practice.

## Simple Summary

Head and neck cancer remains a major health problem, and patients with apparently similar tumors may have very different disease courses and responses to treatment. One reason is the considerable biological variation among these tumors, which cannot be fully understood by examining a single molecular feature. In this review, we discuss studies that combine different types of molecular information to better understand head and neck cancer and identify markers with possible clinical value. The available evidence indicates that tumor development and treatment resistance involve interconnected changes in genes, gene activity, proteins, metabolism, and the cells surrounding the tumor. Recent methods have also provided a more detailed view of the different cell populations present within the same tumor. Despite the large number of potential markers reported, few are currently used in clinical practice. Many findings have not been confirmed in independent groups of patients, and differences between laboratory and analytical methods remain a problem. Further clinical studies using standardized methods are needed to determine which molecular markers can provide useful information for patient care and help doctors make better treatment decisions.

## 1. Introduction

Head and neck cancer (HNC) comprises a heterogeneous group of malignancies arising from the major anatomical subsites, including the oral cavity, oropharynx, hypopharynx, larynx, nasopharynx, nasal cavity, and paranasal sinuses. Head and neck squamous cell carcinoma (HNSCC), which originates from the mucosal epithelium of most of these anatomical sites and accounts for approximately 90% of HNC cases, remains an important cause of cancer-related morbidity and mortality worldwide [1]. Treatment has changed considerably over recent decades, particularly with improvements in surgical techniques, radiotherapy, and the introduction of immunotherapy. Nevertheless, gains in overall survival remain relatively modest. Late diagnosis, disease recurrence, resistance to treatment, and the considerable biological diversity of HNSCC are among the main reasons for these unsatisfactory outcomes [2,3,4]. This heterogeneity is also reflected in the diverse etiological factors associated with these malignancies, including tobacco and alcohol exposure, *human papillomavirus* (HPV) infection, particularly in oropharyngeal squamous cell carcinoma, and *Epstein–Barr virus* (EBV), which is strongly associated with nasopharyngeal carcinoma [1,2,3,4]. Because these anatomical subsites differ substantially in their anatomy, physiology, etiological factors, molecular landscape, tumor microenvironment, and clinical behavior, comprehensive reviews dedicated to individual HNSCC subsites have distinct objectives and scope. In contrast, the present review focuses on molecular features that are shared across HNSCC and that can be investigated through integrative multi-omics approaches, while highlighting site-specific differences whenever supported by the available evidence. Clinical and pathological parameters, including TNM staging, are still central to patient management but provide limited information on the molecular differences that may determine tumor behavior and response to therapy.

The molecular landscape of HNSCC is particularly complex. Alterations involving *TP53*, *CDKN2A*, *EGFR*, and *NOTCH1* coexist with copy number changes, epigenetic abnormalities, and extensive changes in RNA and protein expression [2,5]. The effects of these alterations are not restricted to cancer cells. They also influence the surrounding tumor microenvironment (TME), including its immune and stromal components, and contribute to metabolic adaptation and treatment resistance. The development of high-throughput technologies has made it possible to investigate these molecular features on a much larger scale. However, studies centered on a single molecular layer inevitably show only one part of this complex biological picture.

Genomic studies have been particularly useful for describing the molecular heterogeneity of HNSCC and identifying alterations with possible prognostic value. Still, the presence of a DNA alteration does not necessarily explain the phenotype of a tumor or predict how it will respond to treatment. RNA-based analyses add important information because gene expression changes according to cellular state, pathway activity, and interactions with the immune microenvironment [3]. Epigenetic regulation, especially DNA methylation, also has a relevant role in HNSCC and may improve tumor classification when examined alongside gene expression and copy number alterations [2]. Proteomic and metabolomic studies move the analysis closer to the functional tumor phenotype and have identified changes related to epithelial–mesenchymal transition (EMT), DNA repair, and metabolic reprogramming [6]. Each molecular layer provides distinct but complementary information. While genomic alterations define the molecular background of HNSCC, transcriptomic, epigenomic, proteomic, and metabolomic analyses capture regulatory and functional changes that cannot be inferred from DNA alterations alone [2].

The introduction of single-cell and spatial technologies has added another dimension to the study of HNSCC. Bulk tissue analyses represent an average signal from many cell populations and may therefore conceal biologically relevant differences between individual cells. Single-cell approaches can identify specific cellular populations and their transcriptional programs, whereas spatial analyses preserve information on tissue organization and local cellular interactions within the TME [7]. When different molecular datasets are examined together, additional regulatory and immune-related mechanisms may also become apparent. Some of these integrative studies have already identified molecular patterns associated with immune regulation and response to therapy that were not evident from isolated analyses [5,8].

Despite the large number of biomarkers described in HNSCC, relatively few have reached routine clinical practice. Findings are often difficult to reproduce in independent patient cohorts, and the performance of proposed biomarkers may change considerably between clinical populations. Differences in sample processing, analytical methods, computational pipelines, and patient characteristics further complicate comparison between studies. The limited incorporation of molecular findings into clinical data is another important problem. Taken together, these issues suggest that biomarkers derived from a single molecular level may not be sufficient to represent the biological diversity of HNSCC.

Multi-omics approaches derive their strength from integrating complementary molecular data rather than examining each biological layer independently. This narrative review brings together evidence from genomic, transcriptomic, epigenomic, proteomic, metabolomic, single-cell, spatial, and AI-based studies to provide a comprehensive perspective on biomarker research in HNSCC. Accordingly, the purpose of this review is to critically examine how complementary omics technologies collectively improve our understanding of HNSCC biology and support biomarker discovery and clinical translation across the disease spectrum. Instead of presenting each omics platform separately, we critically examine how these complementary datasets contribute to the understanding of key biological pathways, biomarker performance, and their potential for clinical translation.

## 2. Methods

This is a narrative review. Relevant publications were identified through searches of PubMed, Web of Science, and Scopus conducted in February 2026. The search included combinations of the terms “head and neck squamous cell carcinoma”, “HNSCC”, “genomics”, “transcriptomics”, “epigenomics”, “proteomics”, “metabolomics”, “single-cell sequencing”, “spatial transcriptomics”, “artificial intelligence”, “machine learning”, and “biomarkers”. Original articles, clinical and translational studies, systematic reviews, and meta-analyses. Editorials, letters, conference abstracts, opinion papers, and studies unrelated to molecular biomarkers or multi-omics approaches in HNSCC were excluded. Additionally, studies focused exclusively on technical or computational developments without direct biological or clinical implications were excluded. Full-text articles were reviewed, and the final selection was based on their relevance to the scope of this review and contribution to current knowledge of HNSCC biology and biomarker research. The selected literature was subsequently organized according to the major omics platforms discussed throughout this review.

## 3. Genomic Biomarkers

Genomic biomarkers provide one of the main molecular bases for understanding the biological diversity of HNSCC. Somatic mutations have received most of the attention, but copy number variations (CNVs), loss of heterozygosity (LOH), germline variants, and tumor-derived DNA detected in liquid biopsies are also relevant. Each of these alterations provides different information about tumor development and, in some cases, patient outcome. Somatic mutations are the best characterized and reflect, at least in part, the effects of carcinogenic exposure and the molecular processes occurring during tumor evolution.

Large sequencing studies have identified recurrent mutations affecting tumor suppressor genes, oncogenic pathways, and regulators of the DNA damage response. The mutational profile of HNSCC is now relatively well described, although the clinical meaning of many of these alterations remains uncertain. Their frequency and biological effects may differ according to anatomical site, HPV status, and other tumor characteristics, making direct clinical interpretation difficult.

*TP53* is the most frequently mutated gene across HNSCC subtypes. In high-risk cutaneous squamous cell carcinoma of the head and neck, *TP53* mutations were identified in all tumors analyzed [9]. A high frequency of *TP53* alterations has also been reported in oral squamous cell carcinoma (OSCC), together with mutations in *CDKN2A* and *FAT1* [10]. This pattern is particularly characteristic of HPV-negative tumors, in which loss of tumor suppressor function is a major molecular event. *TP53* alterations are not restricted to tumor tissue. In a saliva-based sequencing study, *TP53* accounted for 80% of the pathogenic variants detected [11]. Thus, *TP53* alterations are consistently found using different biological samples and analytical approaches. However, *TP53* mutation alone has shown limited value for predicting prognosis in clinical practice.

*CDKN2A* alterations are another common feature of HPV-negative HNSCC and contribute to disruption of the RB pathway. HPV-positive carcinomas, on the other hand, have different mutational and copy number profiles and are generally regarded as a distinct biological group [10]. Mutations involving *PIK3CA, PTEN, RB1*, and *CDKN2A* have been reported in p16-positive tumors. Among them, the *PIK3CA* hotspot mutations E542K, E545K, and H1047L lead to persistent activation of the *PI3K/AKT/mTOR* pathway [12]. *PIK3CA* is one of the most frequent oncogenic alterations in HNSCC, but studies evaluating its ability to predict response to targeted treatment have produced variable results. The available evidence also suggests that mutation frequency and clinical utility do not necessarily overlap. *TP53* alterations have been consistently detected across different HNSCC cohorts and biological specimens, yet their prognostic value has remained modest. *PIK3CA* mutations, in contrast, have received greater attention as potential therapeutic targets, although their predictive value appears to vary according to tumor subtype and clinical setting.

Other mutations occur less frequently but may help define specific biological subgroups. *SMAD4* mutations, for example, have been identified in a proportion of HNSCC cases and have been linked to increased tumor cell migration and invasion [13]. Experimental data suggest that these alterations may be more closely related to tumor progression than to initial malignant transformation. Missense mutations in the *MH1* domain can impair the tumor suppressive activity of *SMAD4* and may have prognostic implications, although this association has not yet been sufficiently validated [13].

Genomic studies have also identified molecular differences among HPV-negative tumors. One subgroup of oral cavity carcinomas has few or no copy number alterations and has been described as CNA-quiet. These tumors frequently retain wild-type *TP53* and are enriched for *CASP8* and *HRAS* mutations [14]. They also show distinct clinical and immune characteristics and have been associated with improved overall survival in the cohorts evaluated. This observation suggests that the overall pattern of genomic alterations may provide information that cannot be obtained by examining individual mutations alone. These findings suggest that the overall pattern of genomic alterations may provide information that cannot be obtained by examining individual mutations alone. This interpretation is consistent with studies showing that genomic architecture may have greater prognostic relevance than isolated driver mutations.

Tumor mutations can also be investigated using samples outside of tissue. Next-generation sequencing of plasma and saliva has shown that tumor-derived variants are detectable in circulating cell-free DNA (cfDNA) and salivary DNA. In metastatic OSCC, plasma cfDNA analysis identified mutations in *TTN*, *PLEC*, *SYNE1*, and *USH2A*, as well as recognized cancer-associated genes such as *KMT2D* and *LRP1B* [15]. A higher mutation burden was observed in patients with more advanced disease and metastasis. Salivary DNA analysis detected pathogenic variants in 29.2% of longitudinal samples and, in several cases, identified molecular alterations before locoregional recurrence became clinically apparent [11]. These findings support the potential role of liquid biopsy in disease monitoring. Low variant allele fractions and limitations in analytical sensitivity and specificity, however, remain important obstacles. Despite these limitations, plasma and saliva have produced comparable results for the detection of tumor-derived variants, supporting the use of liquid biopsy as a complementary approach for longitudinal monitoring rather than as a replacement for tissue-based genomic analysis.

Most recurrent somatic alterations in HNSCC involve cell cycle regulation, *TGF-β* signaling, apoptosis, and proliferative pathways. *TP53* and *CDKN2A* affect cell cycle control, *SMAD4* is involved in *TGF-β* signaling, *CASP8* participates in apoptosis, and *PIK3CA* and *HRAS* regulate proliferative signaling. Despite their biological importance, few individual mutations have demonstrated reproducible prognostic or predictive value. Genomic findings may therefore be more informative when considered together with structural alterations, immune characteristics, and longitudinal molecular data.

Structural genomic changes add another level of complexity. Recurrent CNVs in OSCC include gains involving 3q, 5p, 7p, 8q, 11q, and 20q and losses involving 3p, 8p, 9p, and 18q [16]. These changes can increase oncogene dosage or result in the loss of tumor suppressor genes. Amplification of the 9p region containing *MLLT3* has been associated with greater tumor invasiveness and shorter survival [16]. Differences in global copy number patterns may also have prognostic relevance. CNA-quiet tumors, for example, have shown better outcomes than tumors with extensive copy number alterations [14].

LOH is another frequent genomic event in HNSCC and may affect both tumor suppressor function and immune recognition. Loss of one HLA class I allele has been reported in approximately 53% of HNSCC cases and was associated with lower CD8+ T-cell infiltration [17]. Reduced HLA diversity may limit antigen presentation and facilitate immune escape. Chromosome 9p is also a recurrent site of LOH in OSCC and has long been implicated in oral carcinogenesis [18]. CNVs and LOH therefore provide information that complements conventional mutation analysis and may help explain differences in tumor phenotype and immune composition.

Inherited genetic variation also contributes to HNSCC susceptibility. Genome-wide association studies (GWAS) have identified several risk loci, including variants within the HLA region [19]. The *TP53* 3′UTR variant rs78378222 has been associated with a lower risk of HNSCC and may influence *TP53* regulation [19]. Associations involving *BRCA2* and *ADH1B* variants also appear to vary according to tobacco and alcohol exposure. Such findings illustrate how inherited genetic background and environmental carcinogens may interact in determining individual susceptibility to HNSCC [19].

Beyond susceptibility, germline polymorphisms may also influence prognosis and treatment response. Variants in angiogenesis-related genes, including *FGF2*, *PDGFRA*, *PDGFRB*, *MMP2*, and *TIMP2*, have been associated with survival outcomes in patients undergoing radiotherapy or chemoradiotherapy [20]. Similarly, polymorphisms in the lncRNA MALAT1 have been linked to OSCC risk and progression, particularly in individuals exposed to betel quid [21]. Although highly penetrant syndromic predispositions are relatively uncommon in HNSCC, the cumulative effect of low-penetrance variants contributes to polygenic risk and may influence tumor–immune interactions [22].

From a clinical perspective, not all genomic biomarkers have shown the same degree of clinical potential. Recurrent mutations such as *TP53* and *CDKN2A* are valuable for describing the molecular landscape of HNSCC but have shown limited performance as isolated prognostic markers. By comparison, broader genomic features, including CNA burden and HLA loss of heterozygosity, have demonstrated more consistent associations with patient outcome and immune-related characteristics.

**Figure 1:**
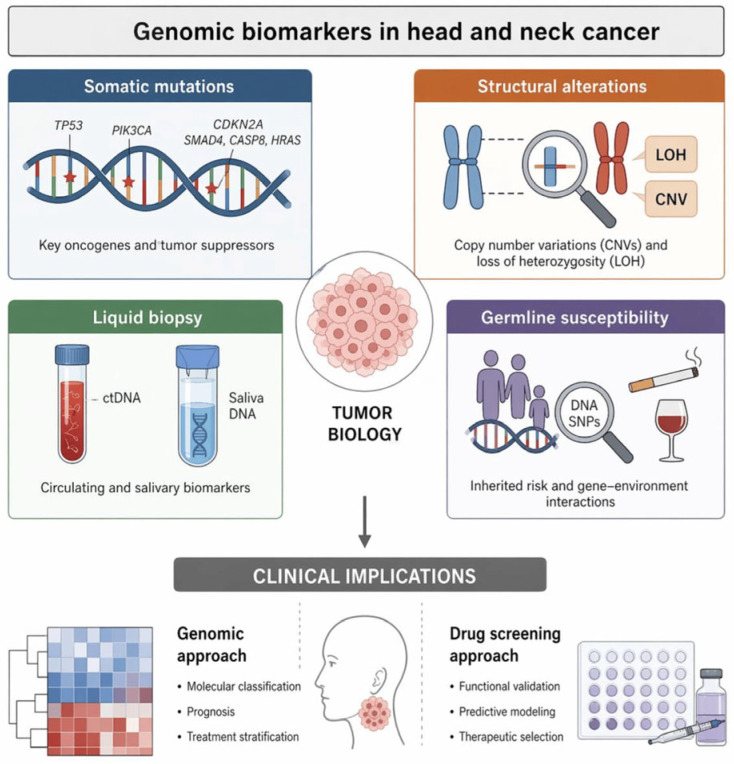
Genomic biomarkers in HNSCC. Somatic mutations, structural genomic alterations (CNVs and LOH), germline susceptibility variants, and liquid biopsy markers provide complementary information on tumor initiation, progression, immune evasion, and disease monitoring. Although most individual alterations remain exploratory biomarkers, combined genomic profiling may support prognostic assessment, patient stratification, longitudinal monitoring, and therapeutic decision-making, pending further clinical validation.

The studies discussed in this section indicate that no single genomic alteration adequately reflects the biological complexity of HNSCC. Markers derived from different genomic levels often provide complementary rather than overlapping information, particularly when structural alterations, inherited variants, and liquid biopsy findings are considered together. Future clinical applications will likely depend on integrative strategies combining somatic mutations, structural alterations, immune-related genomic features, and longitudinal liquid biopsy monitoring (Figure 1).

## 4. Transcriptomic Biomarkers

### 4.1. Gene Expression Profiles Associated with EMT, CSCs, and Tumor Microenvironment

Transcriptomic profiling examines changes in gene expression and can therefore provide information on the biological processes active within a tumor. In HNSCC, expression patterns have been linked to tumor progression, immune activity, and response to treatment. Several studies have identified transcriptional changes involving WNT signaling, DNA repair, histone modification, *TGF-β*, and *MAPK* pathways [23]. Some of these changes may result from genomic alterations, but RNA expression also varies according to cellular state and environmental signals. For this reason, transcriptomic data can provide information on pathway activity that is not always apparent from DNA analysis alone.

Changes in gene expression have also been investigated in relation to treatment response. Chemoradiotherapy appears to affect several immune-related transcriptional programs in HNSCC. In paired tumor samples collected before and after treatment, T-cell-related transcripts were reduced after chemoradiotherapy, particularly in patients who did not achieve a complete response [24]. This reduction may reflect a loss of T cells or changes in their functional state during treatment. The observation is relevant to current attempts to combine chemoradiotherapy with immune checkpoint inhibitors (ICIs), including anti-PD-1 antibodies, although the optimal timing and clinical benefit of these combinations remain under investigation [24].

A substantial proportion of the RNA detected in tumor samples originates from cells in the tumor microenvironment (TME). Analysis of stromal gene expression has identified tumors with predominantly immune, fibroblast, or myeloid components [25]. These patterns were associated with different clinical outcomes. Patients with immune-enriched tumors had better survival than those with fibroblast-rich or myeloid-dominant stroma [25]. Interestingly, stromal characteristics showed a stronger association with outcome than several features of the epithelial tumor compartment. Thus, part of the prognostic information obtained from bulk transcriptomic analysis may reflect differences in the cellular composition of the tumor rather than cancer cell expression alone. This may explain why studies evaluating bulk RNA sequencing have not always identified the same prognostic signatures. Differences in stromal and immune cell composition can substantially influence gene expression profiles, even among tumors with similar epithelial characteristics.

This becomes particularly relevant when epithelial–mesenchymal transition (EMT) is considered. Tumors with EMT-related expression patterns do not necessarily have the same clinical behavior. An EMT phenotype occurring in a fibroblast-rich environment, especially in tumors with abundant cancer-associated fibroblasts (CAFs), has been associated with aggressive disease and poor survival [25]. The outcome is different when a similar EMT program is found in an immune-enriched stroma, where survival is considerably better. This context indicates that the biological consequences of EMT depend, at least in part, on the surrounding cellular environment. Interactions between CAFs and tumor cells may contribute to this process, possibly through *EGFR* signaling and the EGFR ligand AREG [25]. These observations suggest that EMT-associated transcriptional programs should not be interpreted independently of the surrounding microenvironment. Similar EMT signatures may therefore have different biological and clinical implications depending on the cellular composition of the tumor.

Transcriptomic studies have also helped identify the cellular sources of immune checkpoint molecules within HNSCC. PD-L1 expression is frequently associated with macrophage-rich microenvironments, which is relevant to the use of PD-1/PD-L1-directed immunotherapy [26]. Other immune regulatory molecules show different expression patterns. Galectin-9, for example, is found mainly in fibroblasts and other non-immune cells rather than in the same populations expressing PD-L1 [26]. This distinction may be important when considering therapies directed at the TIM-3 pathway. LAG3 expression has also been detected in dendritic cells and macrophages in inflamed tumors and is associated with greater CD8+ T-cell infiltration [26]. The cellular distribution of these molecules therefore needs to be considered when transcriptomic biomarkers are investigated as tools to support the selection of immune-based treatments. The differences observed among these immune checkpoints indicate that transcript abundance alone is unlikely to capture the complexity of the immune response. Their cellular localization may be as relevant as their expression level when evaluating potential biomarkers for immunotherapy.

**Figure 2:**
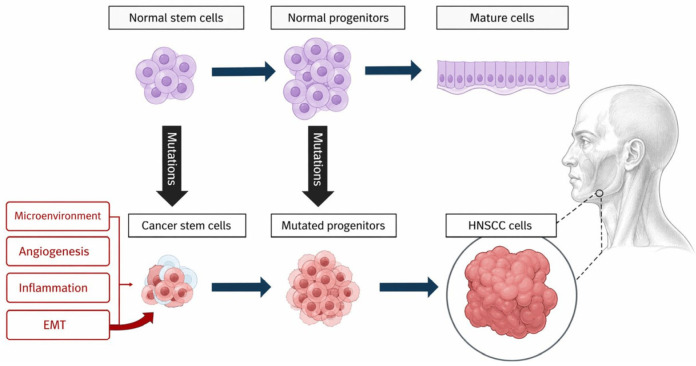
Proposed mechanisms involved in the generation and maintenance of cancer stem cells (CSCs) in HNSCC. Genetic alterations, inflammatory signaling, epithelial–mesenchymal transition, angiogenesis, and interactions with the tumor microenvironment contribute to the acquisition and maintenance of stem-like properties. These processes have been associated with tumor progression, therapeutic resistance, and disease recurrence.

Cancer stem cells (CSCs), also referred to as tumor-initiating cells, are another source of cellular and transcriptional heterogeneity in HNSCC (Figure 2). CSCs have the capacity for self-renewal and differentiation and are frequently associated with resistance to anticancer therapy. Their origin is still debated. They may develop from normal stem or progenitor cells after the accumulation of oncogenic alterations, but differentiated cancer cells can also acquire stem-like characteristics through cellular reprogramming. Genomic instability, inflammatory signals, and conditions within the TME have all been implicated in the development and maintenance of CSC populations [27].

The local environment surrounding CSCs, commonly described as the CSC niche, is important for maintaining these cells. In HNSCC, CSCs are often found close to blood vessels, and experimental studies suggest that signals from tumor-associated endothelial cells contribute to their survival [27]. Depletion or disruption of these endothelial cells reduces CSC populations in experimental models. Inflammation and angiogenesis may also affect the niche, while EMT can promote the acquisition of stem-like properties by tumor cells. These processes are closely interconnected and may explain why transcriptional programs related to stemness are often found in aggressive and treatment-resistant tumors [27].

These findings indicate that transcriptomic signatures are strongly influenced by the cellular composition of the tumor. Integrating bulk RNA data with genomic, spatial, and clinical information may improve biological interpretation by distinguishing tumor-intrinsic programs from signals derived from the surrounding microenvironment.

### 4.2. Signatures Related to Response to Cisplatin, Radiation, and Immunotherapy

Cisplatin resistance is accompanied by considerable changes in the phenotype and gene expression of tumor cells. Resistant OSCC cells often acquire an elongated, spindle-shaped morphology and become more heterogeneous, features commonly associated with a mesenchymal-like phenotype. RNA sequencing of HSC-3R and SCC-9R cells has identified changes in genes involved in extracellular matrix organization, inflammatory signaling, and cellular metabolism [28]. *TNF-α/NF-κB*-related pathways and glycolysis are among the processes altered in these cells. Changes in EMT-related genes are also evident, with increased expression of *ZEB1*, *ZEB2*, and *Vimentin*. Higher levels of *TWIST1*, *N-cadherin*, *MMP-2*, and *MMP-9*, together with reduced *E-cadherin* expression, have also been observed [28]. These molecular and morphological changes are consistent with the acquisition of a more motile and adaptable phenotype during the development of cisplatin resistance. Whether the same expression patterns can reliably predict resistance in patients, however, is still unclear. Although different experimental models have identified alterations involving EMT, inflammatory signaling, and metabolic pathways, the specific genes associated with resistance vary considerably between studies. This suggests that the biological processes involved may be more reproducible than individual transcriptional markers.

Several molecular features have been investigated as possible markers of treatment response in HNSCC. Cisplatin sensitivity has been studied in relation to *TP53* mutations, DNA repair mechanisms, and gene expression patterns identified in resistant tumors. For radiotherapy, HPV and p16 status remain clinically relevant, while transcriptional models such as the Radiation Sensitivity Index (RSI) have been proposed to estimate radiosensitivity. HPV-positive tumors also exhibit distinct immune and transcriptional profiles, typically characterized by increased immune cell infiltration and higher expression of immune-related genes. These differences probably contribute to their greater radiosensitivity, and more favorable clinical outcomes compared with HPV-negative disease.

Expression of chemokines and chemokine receptors, including CXCL2, CCL28, and CCR8, has also been associated with radiation response. Biomarker studies in immunotherapy have largely focused on PD-L1 expression and the combined positive score (CPS), although FOXP3, CTLA-4, CMTM6, tumor mutational burden (TMB), and immune-related expression signatures have also been examined. The performance of these markers differs considerably between studies, and few are sufficiently accurate to guide treatment decisions when considered alone. Among these biomarkers, PD-L1 expression assessed by the combined positive score (CPS) is currently incorporated into clinical decision-making for selected patients, whereas most other transcriptomic signatures remain investigational and require further clinical validation.

Treatment schedule may also affect the balance between therapeutic efficacy and toxicity. In a phase III trial involving patients with high-risk, locally advanced squamous cell carcinoma treated in the postoperative setting, weekly cisplatin-based chemoradiotherapy was non-inferior to cisplatin administered every three weeks [29]. The weekly regimen was also associated with a more favorable toxicity profile. Although these results have practical implications for treatment delivery, molecular markers capable of identifying which patients would benefit most from a particular cisplatin schedule have received much less attention.

Changes in epigenetic regulation have also been described during the acquisition of cisplatin resistance. Exposure to cisplatin can alter the expression of histone deacetylases, including *HDAC1* and *HDAC2*, as well as *SIRT1* and chromatin-associated proteins such as *MTA1* and *BRD4* [30]. Resistant cells may also express higher levels of *BMI*-*1* and KI-67 and show evidence of EMT activation. An inverse association between HDAC1 and *ZEB1* expression has been observed during the development of resistance, with increasing *ZEB1* levels associated with poorer treatment response [30]. Expansion of cancer stem cell (CSC) populations and increased Vimentin expression have been reported in the same context. Epigenetic changes may therefore participate in the transition toward stem-like and mesenchymal cellular states, although the sequence and causal relationship between these events remain to be determined.

Immunotherapy has become an important component of HNSCC treatment. Tumor-associated genomic alterations can generate neoantigens that are recognized by the immune system, but an effective antitumor response depends on the ability of T cells to remain functionally active within the tumor microenvironment. Activation of inhibitory checkpoint pathways can suppress T-cell function after T-cell receptor (TCR) stimulation and allow tumor cells to evade immune control [31]. Most current immunotherapeutic approaches target PD-1/PD-L1, while CTLA-4, LAG3, and TIM-3 are also being investigated as therapeutic targets. Lower CTLA-4 and FOXP3 expression has been associated with a better response to immune checkpoint blockade in some studies [31]. These associations indicate that the cellular and immune composition of the tumor may influence response, but they have not yet resulted in a universally applicable biomarker model. The variability reported between studies probably reflects differences in tumor immune composition rather than the performance of a single biomarker. As a result, current evidence favors the use of combined immune signatures instead of isolated transcriptomic markers.

The clinical use of immunotherapy has improved outcomes for some patients with recurrent or metastatic HNSCC. Reported one-year survival increased from 36% to 57%, while median overall survival increased from 7.7 to 13.0 months following the incorporation of immunotherapeutic approaches [31]. Despite these gains, only a proportion of patients achieve a durable response. Current studies are therefore examining different treatment combinations and biomarkers that may help identify patients more likely to benefit, including those with locally advanced disease.

Although several expression signatures have been associated with treatment response, few have demonstrated sufficient robustness for routine clinical use. Future predictive models will probably depend on integrating transcriptomic data with genomic, epigenetic, and clinical variables rather than relying on isolated expression markers.

### 4.3. Role of Non-Coding RNAs

Changes in non-coding RNA (ncRNA) expression have been described throughout HNSCC development and may also occur in tumors with different responses to treatment [32]. Much of the available evidence concerns microRNAs (miRNAs), although long non-coding RNAs (lncRNAs) and circular RNAs (circRNAs) have received increasing attention. MiRNAs bind to target mRNAs and interfere with post-transcriptional gene regulation; consequently, changes in a single miRNA may affect genes related to proliferation, apoptosis, or invasion. In HNSCC and other cancers, the expression of these molecules is frequently altered. One example is the *miR-21* cluster, which is commonly found at high levels and has oncogenic activity. The opposite pattern is observed for miRNAs with tumor suppressive functions, including *miR-100*, whose expression is often reduced [32]. Rather than acting through an isolated mechanism, these changes affect different targets and signaling networks involved in tumor cell behavior. Different classes of ncRNAs frequently converge on the same biological pathways despite regulating gene expression through distinct mechanisms. This overlap may explain why several studies have identified similar effects on proliferation, invasion, and EMT while implicating different regulatory molecules.

Several long non-coding RNAs (lncRNAs) have been linked to HNSCC biology, particularly through changes in gene expression and cell signaling. These transcripts, conventionally classified as RNAs longer than 200 nucleotides, may act in the nucleus or cytoplasm and their function varies accordingly [32]. Regulation of chromatin and transcription is more commonly attributed to nuclear lncRNAs, while those located in the cytoplasm can interact with mRNAs and alter their stability or translation. Other mechanisms involving miRNA binding and alternative splicing have also been described. Most available evidence has focused on individual lncRNAs, but few candidates have demonstrated reproducible associations across independent cohorts. Functional convergence on common EMT regulators appears to be more consistent than the involvement of any single transcript.

Changes from an epithelial to a mesenchymal phenotype in oral cancer have also been linked to lncRNA expression. Increased E-cadherin and α-catenin levels, together with decreased Vimentin and N-cadherin, were observed after experimental inhibition of EMT-related lncRNAs [33,34]. Among the transcripts studied in this setting are *H19*, *LINC00958*, *NEAT1*, *CILA1*, *HNF1A*-*AS1*, *HOXA11*-*AS*, *KRT16P3*, *SNHG12*, and *MIAT* [33,34]. Some lncRNAs act on transcription factors that control EMT, including SNAIL1, SNAIL2, ZEB1, ZEB2, and TWIST1. In the case of PRKG1-AS1, its downregulation was accompanied by lower SNAIL1 expression [35].

CircRNAs are formed by covalent joining of their RNA ends, resulting in a closed circular structure. This structure increases their stability and resistance to RNA degradation. CircRNAs participate in the regulation of proliferation, migration, invasion, and apoptosis. Different expression patterns have been identified in oral cancer tissues, and several circRNAs have been investigated as candidate diagnostic and prognostic biomarkers and as potential therapeutic targets [32].

NcRNAs are also involved in the response of tumor cells to radiotherapy. Differences in ncRNA expression have been reported between radiosensitive and radioresistant tumors, particularly for molecules that regulate DNA damage repair, apoptosis, and the cell cycle [36]. ATM signaling is one of the pathways involved in the repair of radiation-induced DNA damage. *PVT1* is overexpressed in HNSCC and regulates components of this pathway. In nasopharyngeal carcinoma models, PVT1 downregulation reduced phosphorylation of ATM, p53, and Chk2. These changes impaired DNA repair and increased the response of tumor cells to radiation [36,37].

*MiR-29c* also affects radiosensitivity through the regulation of apoptosis. This miRNA targets members of the Bcl-2 family and promotes apoptotic signaling. Increased *miR-29c* expression enhanced the sensitivity of nasopharyngeal carcinoma cells to radiotherapy in in vitro and in vivo models [36].

Current evidence supports an important regulatory role for ncRNAs in HNSCC biology, but most candidate biomarkers remain supported by experimental or small cohort studies. Larger prospective studies using standardized analytical methods will be necessary before these molecules can be incorporated into clinical biomarker panels.

## 5. Epigenomic Insights

### 5.1. DNA Methylation Mechanisms and Histone Modifications in HNSCC

Changes in DNA methylation and histone regulation are frequently found in HNSCC and affect the expression of genes involved in malignant transformation and tumor progression. In contrast to mutations, these alterations do not modify the nucleotide sequence and may change during tumor evolution or following exposure to treatment. Most studies in HNSCC have examined CpG methylation and enzymes involved in histone modification [38,39].

Both increased and reduced DNA methylation have been reported in cancer. Hypermethylation is often concentrated in CpG-rich promoter regions and can reduce the transcription of tumor suppressor genes. At the same time, loss of methylation across the genome may favor chromosomal instability and abnormal expression of genes that are normally repressed [38]. This combination is found in many malignant tumors, including HNSCC. Some of these changes appear early in carcinogenesis, before the development of advanced disease, and this observation has encouraged the analysis of methylated DNA in studies of tumor detection. Although different studies have identified distinct methylated genes, they have reached similar conclusions regarding the timing of these alterations. Aberrant DNA methylation appears to occur early during tumor development, supporting its investigation as a marker for early detection rather than as a feature restricted to advanced disease. DNA methylation patterns also differ according to HPV status, with HPV-positive tumors showing epigenetic profiles distinct from those of HPV-negative disease, reflecting differences in viral-driven carcinogenesis and host gene regulation.

Histone proteins are also modified during tumor development. Acetyl groups can be added or removed, and histones may undergo methylation, phosphorylation, or ubiquitination [39]. These reactions alter the interaction between histones and DNA and, consequently, the accessibility of transcriptional machinery to specific genomic regions. Histone acetyltransferases (HATs) add acetyl groups, whereas histone deacetylases (HDACs) remove them. Histone methyltransferases (HMTs) regulate histone methylation. Acetylated chromatin is generally more accessible to transcription, although the effect of histone modifications depends on the residue and modification involved. This may partly explain why histone-related biomarkers have shown less consistent results than DNA methylation markers. Different histone modifications can produce distinct biological effects, even within the same signaling pathway, making direct comparisons between studies more difficult.

Abnormal expression or activity of histone-modifying enzymes has been described in HNSCC [38,39]. The resulting transcriptional changes involve cell cycle control, DNA repair, and pathways associated with tumor progression. *TP53* provides one example of the interaction between epigenetic regulation and a major cancer-associated gene. Acetylation of p53 affects protein stability and increases its ability to bind DNA and regulate transcription [38]. Epigenetic alterations have also been reported in tumors with changes in PI3K signaling, indicating that chromatin regulation and oncogenic pathways are not independent events.

The use of epigenetic alterations as biomarkers has been examined in HNSCC, particularly for DNA methylation. Promoter methylation can occur before clinically advanced disease and has therefore been studied in tumor detection and risk assessment [40]. *SEMA3B* promoter hypermethylation has been identified in OSCC and differs between tumor and non-cancerous tissues [39]. Other differentially methylated genes (DMGs), including *PAX9*, *STK33*, *GPR150*, *INSM1*, and *EPHX3*, have been associated with tumor progression or patient outcome [39]. However, most of these markers remain at an exploratory stage, and the biological effects of individual methylation events are not always known. To date, no DNA methylation marker has been incorporated into routine clinical management of HNSCC, although several candidates continue to be evaluated for early detection and prognostic stratification.

Comparison between epigenetic studies is also difficult. Different methods are used for sample processing, methylation detection, and data analysis, and many proposed biomarkers have not been tested in independent patient populations. Histone modifications face similar problems and are less frequently evaluated in clinical cohorts. For these reasons, epigenetic biomarkers are still rarely used in routine management of patients with HNSCC.

### 5.2. Epigenomics as a Prognostic and Predictive Marker

The detection of altered DNA methylation in early stages of carcinogenesis has prompted studies evaluating its use in HNSCC diagnosis and patient assessment [40]. Promoter methylation is of particular interest because transcriptional silencing of tumor suppressor genes may precede advanced tumor development. Methylation profiles have therefore been examined in relation to tumor detection, disease outcome, and differences in treatment response.

Several DNA methylation markers have been investigated as prognostic and predictive biomarkers in HNSCC. Promoter hypermethylation of genes such as *SEMA3B* and differential methylation involving *PAX9*, *STK33*, *GPR150*, *INSM1*, and *EPHX3* have been associated with disease progression or patient outcome [39]. However, these findings have not yet translated into biomarkers suitable for routine clinical decision-making. The variability reported across studies does not appear to reflect a lack of biological relevance but rather differences in study design, patient selection, and analytical methods. Similar limitations have also been observed in other molecular approaches, making external validation an important step before clinical implementation.

Most methylation markers have been identified in retrospective studies or single patient cohorts, limiting their generalizability. Standardized analytical methods and independent prospective validation will be necessary before methylation-based biomarkers can be incorporated into routine clinical practice.

### 5.3. Integration with Other Omics Layers

Integration of epigenomic and metabolomic datasets has provided additional insight into metabolic reprogramming in HNSCC. Metabolomic studies in HNSCC have detected changes in lysoPCs, pyroglutamate, glutamic acid, glucose, arachidonic acid, and choline [41]. Panels containing these metabolites have been investigated for potential applications in tumor detection, risk assessment, and prognosis. Many of the reported metabolites are related to the glucose–alanine and urea cycles or to amino acid metabolism, indicating that several metabolic pathways are altered in HNSCC [41]. Metabolomic data can also be combined with epigenomic and other molecular datasets when constructing multi-omics models. Although the metabolites identified differ between studies, most are linked to pathways involved in energy production, amino acid metabolism, or lipid metabolism. This convergence suggests that metabolic reprogramming is more reproducible than any individual metabolite proposed as a biomarker.

Changes in glucose and lipid metabolism are common during tumor progression. Increased glycolytic activity in HNSCC has been associated with altered expression of *SLC2A1* (GLUT1), *HK1*, *PFKP*, *PKM*, and *LDHA* [41,42]. Increased conversion of glucose to lactate favors its accumulation in the tumor microenvironment and may affect the function of infiltrating immune cells. Lipid metabolism is also altered and contributes to the metabolic requirements of proliferating tumor cells. These metabolic changes have been studied in relation to tumor growth and resistance to treatment. The association between altered glucose metabolism and treatment resistance has been reported in different experimental settings, although the specific metabolic signatures vary according to tumor type, analytical platform, and study design.

The effects of metabolic interference have been examined experimentally. Momordicine-I (M-I), a bioactive compound isolated from Momordica charantia, altered glycolysis and lipid metabolism in HNC models [42]. M-I treatment reduced the expression of metabolic enzymes and induced apoptosis. AMPK activation and reduced mTOR and Akt signaling were also observed [42]. Although these results indicate that tumor metabolism can be pharmacologically modified, evidence for M-I is currently based on experimental models.

Metabolite profiles are strongly influenced by sample type, analytical platform, and the methods used for metabolite identification and quantification. As a result, panels reported in one study may not be reproduced in another cohort. Clinical validation remains limited, and standardized analytical procedures are not yet available for many metabolomic applications. The combination of metabolomic, epigenomic, and other omics data is being explored, but it is still uncertain whether these models provide sufficient additional value for routine clinical use in HNSCC.

## 6. Proteomic and Metabolomic Biomarkers

**Figure 3:**
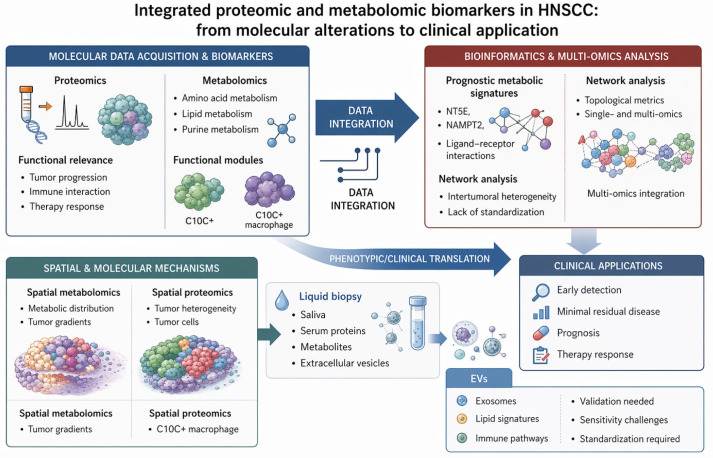
Proteomic and metabolomic biomarkers in HNSCC. Protein and metabolic alterations reflect functional changes associated with epithelial plasticity, immune regulation, metabolic reprogramming, and treatment response. Spatial proteomics and liquid biopsy extend biomarker assessment by capturing intratumoral heterogeneity and enabling longitudinal, minimally invasive molecular monitoring.

Proteomic studies have identified changes in proteins expressed by tumor cells and other cellular populations within HNSCC. Unlike DNA alterations, protein abundance can vary according to cellular activity and conditions in the tumor microenvironment (Figure 3) [43]. This has led to the investigation of protein profiles in relation to tumor progression, treatment response, and patient outcome.

*ARPC4* is one of the immune-related proteins reported at higher levels in OSCC and has been associated with shorter survival [44]. Its expression has also been linked to C1QC-positive macrophages, a population found within the tumor microenvironment. Other proteins provide information on tumor cell phenotype. *KRT17*, for example, is associated with epithelial differentiation and cellular plasticity and has been studied during malignant transformation and phenotypic changes in tumor cells [45]. Although numerous proteins have been associated with clinical characteristics of HNSCC, results for individual markers often differ between patient cohorts. This variability contrasts with the greater consistency observed at the pathway level. Although individual proteins are not always reproduced across studies, many are involved in biological processes related to epithelial plasticity, immune regulation, and tumor progression.

Metabolite profiles also change during HNSCC progression. Alterations in purine, amino acid, and lipid metabolism have been reported and may affect both tumor cells and the surrounding microenvironment. Some studies have combined metabolomic data with transcriptomic or single-cell analyses to examine these changes in specific cellular populations [46]. Models based on ligand–receptor interactions have identified *NT5E* and *AMIGO2* among metabolic regulators associated with immune cell infiltration and response to immunotherapy [46]. Whether these metabolic signatures can be reproduced in independent cohorts remains uncertain. As observed for proteomic biomarkers, the biological pathways identified are generally more consistent than the individual metabolites included in each signature. Differences in analytical platforms and patient populations probably contribute to this variation.

The distribution of metabolites within a tumor is not uniform. Mass spectrometry imaging can detect metabolites directly in tissue sections and show differences between individual tumor regions [47]. This approach has been used to examine local metabolic changes and gradients within the tumor microenvironment. Spatial proteomic methods similarly map protein expression while preserving tissue architecture and can distinguish molecular differences between histologically defined areas [47]. These technologies provide information that is lost when the entire tumor is analyzed as a single sample. This additional spatial information may partly explain why biomarkers identified by imaging-based approaches do not always coincide with those obtained from conventional bulk analyses. Rather than representing conflicting results, these methods often capture different aspects of tumor biology.

Proteins and metabolites have also been evaluated together in clinical biomarker studies. Analyses combining serum protein and metabolite measurements have been evaluated for predicting treatment response, survival, and therapy-associated toxicity [48]. In some studies, combined models performed better than models derived from a single data type. Larger multi-omics studies are now incorporating proteomic and metabolomic measurements with computational methods, including artificial intelligence, for cancer detection and assessment of minimal residual disease [49]. The sensitivity and reproducibility of these models still require evaluation in independent clinical populations.

Saliva is another source of proteins and metabolites in HNSCC. Because tumors of the oral cavity and adjacent head and neck sites may release molecular components into saliva, this fluid has been investigated for cancer detection and disease monitoring [50]. Salivary samples contain proteins, metabolites, extracellular vesicles, and nucleic acids. Differences in sample collection and processing, however, can substantially affect the molecules detected and remain an important issue in biomarker studies.

Extracellular vesicles isolated from saliva have recently been analyzed using proteomic and lipidomic methods. Changes involving immune-related proteins and lipid metabolism have been reported in HNSCC [51]. Specific lipid profiles identified in salivary exosomes have also been evaluated for their ability to distinguish patients with cancer from control individuals. Most available studies are relatively small, and the specificity of these signatures for HNSCC has not been established.

Proteomic and metabolomic analyses measure molecular changes that occur downstream of genomic and transcriptional regulation and may therefore add information on the phenotype of tumor cells and their microenvironment. At present, differences in analytical platforms, sample preparation, and data processing limit comparison between studies. To date, no proteomic or metabolomic biomarker has been incorporated into routine clinical management of HNSCC. Most candidates remain investigational despite encouraging associations with prognosis and treatment response. Protein and metabolite biomarkers will require validation in independent cohorts and assessment alongside established clinical variables before their contribution to HNSCC management can be determined. Current evidence indicates that the main challenge is not the identification of candidate proteins or metabolites, but the reproducibility of these signatures across different analytical platforms and independent patient cohorts.

## 7. Single-Cell and Spatial Omics

Bulk molecular analyses of HNSCC measure signals derived from malignant, immune, and stromal cells within the same sample. As a consequence, transcriptional programs restricted to specific cell populations may be difficult to identify. Information on the position of these cells within the tissue is also lost. Single-cell and spatial omics were developed to examine molecular differences at cellular resolution and, in the case of spatial methods, retain information on tissue organization [52,53].

Studies using single-cell transcriptomics have shown considerable variation among malignant cells from the same HNSCC tumor. Expression programs related to cell proliferation, metabolism, oxidative stress, and invasion have been identified in different tumor cell populations [54,55]. These transcriptional states are not necessarily restricted to separate tumors, and more than one program can be detected within an individual lesion. Tumor cells may also move between cellular states in response to changes in their environment or treatment. Such variation within the malignant compartment may partly explain why a single tumor contains cell populations with different biological properties and treatment sensitivities. These observations help explain why biomarkers identified in bulk analyses are not always reproduced when evaluated at single-cell resolution. Expression differences previously attributed to tumors may instead reflect changes in the proportion of specific cellular populations.

The immune and stromal compartments show similar cellular diversity. Single-cell studies have identified cytotoxic, regulatory, and exhausted T-cell populations in HNSCC, together with myeloid cells displaying inflammatory or immunosuppressive expression profiles [56,57]. Cancer-associated fibroblasts (CAFs) can also be separated into populations with different transcriptional characteristics. Some are associated with extracellular matrix production, whereas others show expression patterns related to immune regulation or paracrine signaling. The proportion and distribution of these populations differ between tumors and may influence the molecular profile obtained from the tissue as a whole. The influence of stromal composition on molecular profiling has also been reported in bulk transcriptomic studies, reinforcing the need to interpret expression signatures in the context of tissue cellularity.

Integrative studies combining single-cell transcriptomics with genomic and clinical data have further refined tumor characterization. For example, molecular features such as mutational burden and immune infiltration can be linked to gene expression patterns at cellular resolution, as demonstrated for genes such as *GSDMB*, which is associated with immune activity, drug sensitivity, and prognosis in OSCC [58]. Similarly, multi-omics pipelines incorporating machine learning have identified biomarkers such as *SASH1*, whose expression is restricted to specific cellular populations and spatial niches [7].

While single-cell approaches provide detailed information on cellular heterogeneity, they lack spatial context. Spatial omics technologies overcome this limitation by preserving tissue architecture and enabling localization of gene expression patterns within tumors [52]. In HNSCC, spatial transcriptomic analyses have demonstrated that aggressive transcriptional programs are not uniformly distributed but are enriched in specific anatomical regions, particularly at tumor–stroma interfaces and invasive fronts. This spatial organization is consistent with previous observations linking EMT, immune regulation, and stromal remodeling to tumor progression, but also demonstrates that these processes are confined to specific tumor regions rather than uniformly distributed throughout the lesion.

Spatially resolved analyses also reveal that stromal and immune components are organized into structured niches rather than randomly distributed populations. Distinct CAF subtypes and immune cells frequently colocalize with specific tumor cell states, forming functional microenvironments that promote immune evasion, oxidative stress adaptation, and tumor progression [56]. Integration of spatial and single-cell data enables more accurate inference of cell–cell communication networks, linking ligand–receptor interactions to defined anatomical regions.

Further integration of single-cell, spatial, genomic, and proteomic data has enabled the identification of key regulatory drivers of tumor progression. For instance, *PAK2* has been identified as a central regulator linking programmed cell death, CSC properties, and immune modulation, with spatial expression patterns highlighting its role within specific tumor niches [59]. In addition, studies combining exome sequencing with single-cell and spatial transcriptomics have shown how etiological factors, such as betel quid exposure, reshape both the genomic landscape and the tumor microenvironment, altering intercellular communication networks and promoting aggressive phenotypes [54].

Assigning a molecular signal to the cell population from which it originates is important when evaluating biomarkers in HNSCC. A marker detected in bulk tissue may derive from malignant cells, fibroblasts, or infiltrating immune cells, and its interpretation can change according to its cellular source. Single-cell data can help resolve this issue, while spatial methods show where marker-expressing cells are located within the tumor. Studies combining bulk, single-cell, and spatial datasets have also used machine learning to compare molecular signatures across these different data types [7]. Signatures initially identified by single-cell analysis can subsequently be mapped to specific tissue regions, allowing their association with local histological features and tumor behavior to be examined.

Despite current technical and analytical limitations, the principal contribution of single-cell and spatial omics is the identification of the cellular origin and spatial context of molecular biomarkers, providing biological information that cannot be obtained from bulk analyses alone.

## 8. Multi-Omics Integration and AI/ML Approaches

**Figure 4:**
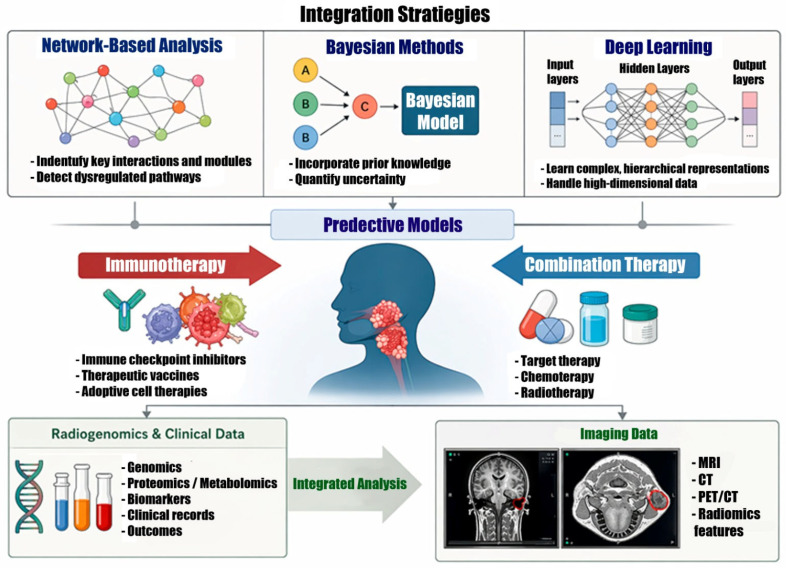
Integration of multi-omics data through AI/ML approaches in HNSCC. Computational models integrate molecular, imaging, and clinical data with the potential to improve biomarker discovery, therapeutic response prediction, and patient stratification. Radiogenomics illustrates how imaging features can be linked to molecular alterations while highlighting the challenges of validation and clinical implementation.

Genomic, transcriptomic, epigenomic, proteomic, and metabolomic alterations are usually analyzed as separate datasets, although changes at one molecular level may affect several others. Multi-omics studies combine these measurements to examine relationships between molecular events and identify patterns that are not apparent when each dataset is evaluated independently (Figure 4). This type of analysis has been applied to HNSCC to investigate tumor heterogeneity and differences in disease progression or treatment response [7].

Combining molecular datasets creates analytical problems because the number and type of variables differ considerably between omics platforms. Network analysis, Bayesian models, machine learning (ML), and artificial intelligence (AI) have been used to handle these data. Bayesian approaches allow previous biological information to be included in the model and provide estimates that account for uncertainty. These characteristics can be useful in cancer studies, in which sample numbers are often small in relation to the number of molecular variables and missing or noisy measurements are common.

One of the most promising applications of multi-omics integration combined with machine learning is the development of predictive models for therapeutic response. Beyond molecular data, AI has also been applied to clinical workflows, particularly in screening and diagnosis. Bayesian deep learning models have been proposed as decision-support tools capable of triaging patients in resource-limited settings. For instance, a model based on the VGG19 architecture combined with Monte Carlo dropout achieved an accuracy of approximately 85.6% in classifying oral lesions, while also providing uncertainty estimates that improved diagnostic reliability when integrated into referral strategies [60]. The ability to quantify predictive uncertainty represents an important advantage over conventional models, as it allows identification of cases requiring expert evaluation. Although these models have shown encouraging results, differences in patient populations and image acquisition protocols have limited direct comparison between studies. Their clinical performance therefore remains uncertain outside the cohorts used for model development.

In medical imaging, machine learning approaches have significantly advanced tumor detection and characterization. Convolutional neural networks have enabled automated feature extraction and segmentation from imaging modalities such as CT, MRI, and PET. However, model performance remains sensitive to hyperparameter optimization and dataset characteristics. Recent approaches, such as Bayesian Optimization-derived Scheduling (BOS), have improved model performance by optimizing training parameters, achieving competitive segmentation accuracy in HNSCC imaging datasets [61]. Nevertheless, limitations such as small sample sizes, lack of external validation, assay optimization and standardization, reproducibility across independent cohorts, regulatory approval, and demonstration of cost-effectiveness remain significant barriers to the clinical implementation of multi-omics biomarkers.

The integration of imaging and molecular data has led to the emergence of radiogenomics, which seeks to correlate quantitative imaging features with underlying genomic and transcriptomic profiles. Radiomic analyses can capture tumor heterogeneity through features related to shape, texture, and intensity, while radiogenomic approaches aim to infer molecular characteristics non-invasively. In HNSCC, radiomic signatures have shown associations with key molecular alterations, including *TP53* and *CDKN2A*, as well as with immune-related features such as CD8+ T-cell infiltration and PD-L1 expression [48]. However, these associations are not consistently reproducible, and predictive performance for clinical outcomes, particularly response to immunotherapy, remains limited. These findings illustrate a recurring pattern in multi-omics research. Associations between imaging features and molecular alterations are biologically plausible, but their reproducibility across independent datasets has been more difficult to demonstrate.

The clinical use of AI-based multi-omics models is currently limited by differences in data acquisition, preprocessing, and analytical procedures. Molecular measurements obtained from different platforms are not always directly comparable, and many studies include relatively small patient cohorts. Interpretation is another problem, particularly for machine learning models in which the contribution of individual variables to a prediction is difficult to determine. Most available models have also been developed and tested using retrospective datasets, and their performance in other patient populations is not well established.

AI methods are increasingly being explored to combine molecular datasets and identify patient groups or biomarkers associated with clinical outcomes. Whether these models improve treatment selection over established clinical and molecular variables remains uncertain. Prospective testing, external validation, and clearer reporting of model development and interpretation are needed before AI-based multi-omics models can be incorporated into routine management of patients with HNSCC.

## 9. Clinical Perspectives

Clinical and molecular variables are increasingly being analyzed together in HNSCC studies using artificial intelligence and machine learning (AI/ML). Genomic and other omics measurements can be combined with imaging and patient data to construct models for prognosis, treatment response, and patient classification. Most of these models, however, have been developed in retrospective cohorts, and differences in analytical methods and study populations make their performance difficult to compare. This limitation has been observed regardless of the molecular platform evaluated. Although different studies have proposed distinct biomarkers and predictive models, relatively few have demonstrated reproducible performance across independent clinical cohorts. A summary of the principal biomarkers discussed in this review, including their omics platform, biological sample, proposed clinical relevance, current level of clinical implementation, major limitations, and representative references, is provided in Supplementary Table S1.

Prediction of response to immune checkpoint inhibitors (ICIs) is a primary application being investigated. The models used for this purpose have been classified as empirical, algorithmic, or mechanistic [62]. Empirical models are based on biomarkers already used or widely investigated in immunotherapy, including PD-L1 expression, tumor mutational burden (TMB), and microsatellite instability (MSI). None of these markers consistently identifies all patients who respond to ICIs. Algorithmic models use clinical and molecular variables as inputs for machine learning methods and generate a score or predicted outcome. Mechanistic models differ in that they attempt to reproduce biological interactions between tumor and immune cells. Many models show good performance in the datasets used for their development but perform less consistently when tested in independent patient populations [62]. Despite their methodological differences, empirical, algorithmic, and mechanistic models share the objective of improving patient selection. Current evidence suggests that model validation has become a greater challenge than biomarker discovery itself.

Immune cell composition has also been included in predictive models. Tumors with higher numbers of CD8^+^ cytotoxic T cells, memory CD4^+^ T cells, and plasma cells have been associated with better responses to immunotherapy. High TMB and expansion of tumor-reactive T-cell clones have also been linked to clinical outcome. These variables describe different aspects of the antitumor immune response and may provide different information when analyzed in the same model.

Functional testing provides another approach to treatment response. Patient-derived organoids (PDOs) have been used to examine the sensitivity of individual tumors to chemotherapy and targeted agents, including anti-EGFR therapies [43]. Organoids retain several characteristics of the tumor from which they are derived and allow drugs to be tested directly in an experimental system. They are also being evaluated as a method for examining candidate biomarkers identified from molecular studies. The extent to which organoid response reproduces clinical response in patients remains an active area of investigation.

Multi-omics studies combine genomic, transcriptomic, epigenomic, proteomic, and metabolomic measurements in the same analytical framework. Single-cell data can be added to determine which cellular populations express a molecular marker, while spatial methods show the location of these populations in tumor tissue. Such information may help distinguish signals derived from malignant cells from those produced by immune or stromal components. This distinction is particularly important for biomarkers identified from bulk tumor samples.

Imaging data have also been incorporated into molecular models. Radiogenomic studies compare radiomic features obtained from medical images with genomic or other molecular measurements and have been used to investigate tumor phenotype and treatment response [48]. Results differ between studies, partly because image acquisition, segmentation, and feature extraction are not standardized. Many reported models have not undergone prospective testing or external validation.

Data integration becomes more difficult when information is collected at different institutions or generated using different platforms. Sample size is another concern because the number of molecular variables may greatly exceed the number of patients included in a study. Privacy requirements can further restrict the transfer of clinical and molecular data between centers. Federated learning has been proposed as one method to address the latter problem by allowing models to be trained using data from multiple institutions without centralizing individual patient records [55].

Prospective evidence remains limited. Multi-omics and AI-based analyses are beginning to be incorporated into clinical studies, including trials of neoadjuvant immunotherapy, but evidence of improvement in survival or reduction in treatment toxicity is not yet available for most models. Cost-effectiveness has also received little investigation. Large multicenter cohorts and prospective testing will be necessary to determine whether a model developed from complex molecular datasets provides clinically useful information beyond established prognostic and predictive factors.

The studies reviewed here indicate that integrating molecular, cellular, imaging, and clinical data has the potential to improve biomarker performance in HNSCC. However, the complexity of these models also increases the need for standardized analytical methods, external validation, and prospective clinical evaluation. Ultimately, their clinical relevance will depend less on the number of molecular layers included than on their ability to provide information that complements existing prognostic and predictive tools.

## 10. Future Directions

The next stage of multi-omics biomarker research will require testing in larger and independent patient populations. Prospective multicenter studies are particularly important because they allow molecular models to be evaluated under different clinical and technical conditions. Clinical, pathological, and imaging variables should also be considered when assessing the additional value of a molecular signature. Radiogenomic analysis and liquid biopsy may be useful in this setting because they allow tumor characteristics to be examined without repeated tissue sampling and, in the case of liquid biopsy, at different points during treatment.

AI/ML provide methods for analyzing datasets containing large numbers of molecular and clinical variables. Their use does not, however, resolve problems related to poor data quality or inadequate validation. Complex models may be difficult to interpret, and the biological basis of a prediction is not always evident. Explainable AI methods are being developed to identify variables that contribute to model predictions. Federated learning offers another approach for multicenter studies by allowing model training across institutions without transferring individual patient data to a central database.

Treatment selection is one of the areas in which integrated molecular data are being investigated. Immune, metabolic, and epigenetic measurements may provide information on different mechanisms of treatment resistance and response. Their combined analysis has been proposed for predicting immunotherapy response and identifying pathways that could be targeted in combination treatments. At present, evidence that these models improve therapeutic decisions compared with established clinical biomarkers remains limited.

## 11. Conclusions

The molecular profile of HNSCC cannot be fully described by a single type of bio-logical measurement. Genomic alterations, changes in RNA and protein expression, epigenetic regulation, and tumor metabolism are interconnected, although they have traditionally been examined in separate studies. The analysis of these datasets together has identified molecular patterns associated with tumor progression, treatment resistance, and immune activity. Single-cell and spatial methods have added information on the cellular origin and tissue distribution of some of these molecular signals. The number of biomarkers identified from multi-omics studies is considerably greater than the number that has reached clinical evaluation. Currently, only a limited number of biomarkers, most notably PD-L1 expression for immunotherapy selection, have been incorporated into routine clinical practice, whereas the vast majority of ge-nomic, transcriptomic, epigenomic, proteomic, and metabolomic biomarkers remain investigational despite encouraging results in retrospective and early clinical studies.

Multi-omics studies have expanded the molecular information available for HNSCC, but the clinical value of an integrated model cannot be determined from molecular complexity alone. A clinically useful biomarker should reproduce its performance in independent populations, demonstrate clinical utility beyond established prognostic factors, and provide information that meaningfully influences patient assessment or treatment selection. Standardized methods and prospective evaluation are therefore needed before multi-omics models can be incorporated into routine HNSCC care. The major challenge is no longer generating increasingly complex molecular datasets, but identifying combinations of biomarkers that are reproducible, clinically interpretable, and capable of improving patient management beyond established prognostic factors.

## Data Availability

No new data were created or analyzed in this study. Data sharing is not applicable to this article.

## Supplementary Materials

The following supporting information can be downloaded at: https://www.mdpi.com/article/10.3390/biology15151321/s1, Table S1: Summary of the main multi-omics biomarkers investigated in HNSCC and their current clinical status.

## Abbreviations

The following abbreviations are used in this manuscript:

BOS	Optimization-derived Scheduling
CAFs	Cancer-Associated Fibroblasts
CDKN2A	Cyclin-Dependent Kinase Inhibitor 2A
cfDNA	Circulating Cell-Free DNA
CNVs	Copy Number Variations
CSCs	Cancer Stem Cells
CTLA-4	Cytotoxic T-Lymphocyte–Associated Protein 4
EGFR	Epidermal Growth Factor Receptor
EMT	Epithelial–Mesenchymal Transition
GWAS	Genome-Wide Association Studies
HATs	Histone Acetyltransferases
HDACs	Histone Deacetylases
HLA	Human Leukocyte Antigen
HMTs	Histone Methyltransferases
HNC	Head and Neck Cancer
HNSCC	Head and Neck Squamous Cell Carcinoma
ICIs	Immune Checkpoint Inhibitors
lncRNAs	Long Non-Coding RNAs
LOH	Loss of Heterozygosity
MAPK	Mitogen-Activated Protein Kinase
M-I	Momordicine-I
miRNAs	MicroRNAs
ML	Machine Learning
mRNA	Messenger RNA
ncRNAs	Non-Coding RNAs
NOTCH1	Notch Receptor
OSCC	Oral Squamous Cell Carcinoma
PD-1	Programmed Cell Death Protein 1
PD-L1	Programmed Death-Ligand 1
PDOs	Patient-Derived Organoids
PI3K	Phosphoinositide 3-Kinase
RSI	Radiation Sensitivity Index
TCGA-HNSC	The Cancer Genome Atlas Head and Neck Squamous Cell Carcinoma
TCR	T-Cell Receptor
TGF-β	Transforming Growth Factor Beta
TMB	Tumor Mutational Burden
TME	Tumor Microenvironment
TP53	Tumor Protein p53
