# Supplementary material for: Multi-Omics Biomarkers in Head and Neck Squamous Cell Carcinoma: Biological Insights and Clinical Translation: A Narrative Review
Source: Biology (Basel). 2026 Aug 6;15(15):1321. doi: 10.3390/biology15151321 (PMC13463855; doi:10.3390/biology15151321)
Supplement: Supplementary file 1 [file biology-15-01321-s001.zip › biology-4450400-supplementary.pdf]

Supplementary Table S1. Summary of the main multi-omics biomarkers investigated in HNSCC and their current clinical status.

Omics platform	Main biomarker(s)	Sample type	Anatomical subsite	HPV/EBV status	Proposed clinical relevance	Clinical implementation	Major limitations	Key reference(s)
**Genomics**	TP53	Tumor tissue, saliva	Cutaneous SCC of the head and neck; Oral cavity; Mixed HNSCC	Mixed HPV status [10, 11]; Not reported [9]	Molecular classification; prognosis	Exploratory	Limited prognostic value as an isolated biomarker	[9–11]
	PIK3CA	Tumor tissue	Mixed HNSCC	Mixed HPV status	Therapeutic target; prognosis	Exploratory	Variable predictive value	[10, 12]
	CDKN2A	Tumor tissue	Mixed HNSCC	Mixed HPV status	Molecular classification	Exploratory	Limited predictive utility	[10]
	HLA LOH	Tumor tissue	Mixed HNSCC	Not reported	Immune evasion; prognosis	Exploratory	Requires prospective validation	[17]
	CNA burden / CNA-quiet subtype	Tumor tissue	Mixed HNSCC; predominantly OSCC	HPV-negative [14]; Not reported [16]	Prognosis	Exploratory	No standardized thresholds	[14, 16]
	cfDNA / salivary DNA	Plasma, saliva	Mixed HNSCC; predominantly OSCC	Mixed HPV status [11]; Not reported [15]	Disease monitoring	Exploratory	Low analytical sensitivity	[11, 15]

**Transcriptomics**	EMT-related signature	Tumor tissue	Mixed HNSCC; predominantly OSCC	HPV-negative [28,30]	Cisplatin resistance; prognosis	Exploratory	Considerable inter-study variability	[28, 30]
	PD-L1 CPS	Tumor tissue	Mixed HNSCC	Mixed HPV status	Immunotherapy selection	Clinical practice	Limited predictive accuracy	[26]
	Radiation Sensitivity Index (RSI)	Tumor tissue	Mixed head and neck cancer	Not reported	Prediction of radiosensitivity	Investigational	Limited external validation	[29]
	Immune-related gene signatures	Tumor tissue	Mixed HNSCC	Mixed HPV status [24-26]	Prognosis; immunotherapy response	Exploratory	Lack of standardized signatures	[24–26]
	ncRNAs (miR-21, miR-100, H19, NEAT1, PVT1, etc.)	Tumor tissue	Mixed HNSCC; predominantly OSCC	HPV-negative	Diagnosis; prognosis; therapy response	Exploratory	Mostly experimental studies	[37]
**Epigenomics**	DNA methylation (SEMA3B, PAX9, STK33, GPR150, INSM1, EPHX3)	Tumor tissue	Mixed head and neck cancer	Mixed HPV status [39]; Not reported [40]	Early detection; prognosis	Exploratory	No clinically implemented biomarkers	[39, 40]
	Histone modifiers	Tumor tissue	Mixed head and neck cancer	Mixed HPV status [39];	Treatment response	Experimental	Limited clinical validation	[38–40]

	(HDACs, HATs, HMTs)			Not reported [38, 40]				
**Metabolomics**	LysoPCs, glutamate, arachidonic acid, choline	Tissue, saliva	Mixed head and neck cancer	Not reported	Diagnosis; metabolic profiling	Exploratory	Platform-dependent variability	[42]
**Single-cell / Spatial omics**	CAF subsets	Tumor tissue	Mixed HNSCC	Not reported [44]; HPV-negative [45]	Prognosis; therapeutic targets	Exploratory	High cost and analytical complexity	[44–45]
	Exhausted CD8+ T cells	Tumor tissue	Mixed HNSCC	Not reported [44]; HPV-negative [45]	Immunotherapy response	Exploratory	Lack of standardized pipelines	[44–45]
	Spatial immune architecture	Tumor tissue	Mixed HNSCC	Not reported [44]; HPV-negative [45]	Patient stratification	Exploratory	Limited prospective validation	[44–45]
**Multi-omics / AI**	Integrated multi-omics models	Integrated datasets	Mixed HNSCC	Not reported [47-49,51]	Prognosis; treatment response	Exploratory	Overfitting, data leakage, limited external validation	[47–49, 51]

Abbreviations: AI, artificial intelligence; CAF, cancer-associated fibroblast; cfDNA, circulating cell-free DNA; CNA, copy number alteration; CPS, combined positive score; EMT, epithelial–mesenchymal transition; HLA LOH, human leukocyte antigen loss of heterozygosity; HMT, histone methyltransferase; HAT, histone acetyltransferase; HDAC, histone deacetylase; ncRNAs, non-coding RNAs; RSI, Radiation Sensitivity Index.
